# Low grade of satisfaction related to the use of current systemic therapies among pustular psoriasis patients: a therapeutic unmet need to be fulfilled

**DOI:** 10.3389/fmed.2023.1295973

**Published:** 2024-01-11

**Authors:** Giulia Coscarella, Gennaro Marco Falco, Gerardo Palmisano, Elena Ippoliti, Eleonora De Luca, Niccolò Gori, Lucia Di Nardo, Giacomo Caldarola, Clara De Simone, Andrea Chiricozzi, Ketty Peris

**Affiliations:** ^1^Dermatologia, Dipartimento Scienze Mediche e Chirurgiche, Fondazione Policlinico Universitario A. Gemelli IRCCS, Rome, Italy; ^2^Dermatologia, Dipartimento Universitario di Medicina e Chirurgia Traslazionale, Università Cattolica del Sacro Cuore, Rome, Italy

**Keywords:** psoriasis, pustular, patient satisfaction, quality of life, biologics

## Abstract

**Introduction:**

Pustular psoriasis is considered a separate entity from plaque psoriasis and can be categorized as generalized pustular psoriasis (GPP), acrodermatitis continua of Hallopeau, or palmoplantar pustulosis (PPP). Current guidelines mostly include treatment options that have not been specifically developed for the treatment of pustular psoriasis. The majority of them does not have indication for the treatment of pustular psoriasis. Their effectiveness and safeness have been described in small cohort-based studies or case series with a low level of evidence. Previous studies evaluated treatment response through physician-based assessment but none reported patient satisfaction to treatment, quality of life and patient perception of disease severity during systemic therapies, particularly with biologics commonly used in plaque psoriasis. This study aimed to investigate patient satisfaction to treatment and patients’ quality of life during treatment, correlating patient-reported outcomes with residual disease severity.

**Methods:**

A cross-sectional, cohort-based, single center study included patients affected by pustular psoriasis undergoing treatment with systemic agents. Demographic, clinical characteristics were collected. Treatment satisfaction as well as disease severity were assessed through dedicated assessment scores.

**Results:**

A total of 31 patients affected by GPP or PPP were included. Despite biologic treatment, 80.6% of patients continued to experience mild-to-severe disease activity, with discrepancies between patient and physician assessments. Patients reported a substantial impairment in their quality of life, with notable limitations in physical activity and emotional distress. Mental health conditions, such as depression and anxiety disorders, were common. Treatment satisfaction varied, with moderate scores for effectiveness and convenience. Only a small proportion of patients (41.9%) reported complete or high overall treatment satisfaction. GPP and PPP subcohorts exhibited similar quality of life and treatment satisfaction levels.

**Discussion:**

This study highlights the suboptimal control of PP despite biologic therapies, resulting in a significant impact on patients’ quality of life and treatment satisfaction. The findings highlight the need for specific therapies and standardized guidelines for managing PP. New targeted therapies, such as spesolimab, hold promise for optimizing treatment satisfaction and improving patients’ quality of life in this challenging condition. Future research should focus on refining treatment strategies to address the unmet needs of PP patients comprehensively.

## Introduction

Psoriasis is a chronic inflammatory skin disease resulting from a genetic predisposition and environmental factors that trigger the development of the psoriasis phenotype. It equally affects men and women (1). Despite psoriasis can be categorized in different clinical subtypes, pustular psoriasis is being recently considered as separate entity (2). According to the European Rare and Severe Psoriasis Expert Network (ERASPEN), pustular psoriasis is a chronic neutrophilic skin disease that includes 3 clinical variants: generalized pustular psoriasis (GPP), characterized by the development of sterile pustules diffused on non-acral skin, palmoplantar pustulosis (PPP) with pustules on palms and/or soles, and Acrodermatitis continua of Hallopeau, characterized by persistent, sterile pustules affecting the nail apparatus (3–5).

Generalized pustular psoriasis (GPP) may coexist with plaque psoriasis and both can be associated with systemic inflammation and symptoms (2).

Generalized pustular psoriasis (GPP) is a rare condition and its prevalence varies between countries ranging from 0.01 to 0.05% (6). A survey from 121 clinics in France estimated disease prevalence around 1.76 case per million people (7), while findings from a Japanese study, collecting data from 575 hospitals, indicated a prevalence of approximately 7.46 per million for GPP (8). A higher prevalence was observed in Germany, with 23.7 case per million assumed (9), and South Korea, counting for 88 to 124 cases per million (10).

The most common precipitating factors of GPP flares include emotional stress, steroid withdrawal, and infections (6). Typically, precipitating factors are considered responsible for about 85% of episodes of GPP, while in almost 15% of patients, flares occur as spontaneous episodes (11, 12).

Limited knowledge on underlying mechanism and the exact pathogenesis of GPP is available. Loss-of-function mutations in IL36N are considered the main cause of GPP, being detected in 23 and 37% of familial and sporadic cases, respectively (13, 14).

The partial understanding of GPP pathogenesis did not facilitate the identification of potential druggable targets, thus, the currently available therapeutic options have not been specifically developed for the treatment of pustular psoriasis, except for spesolimab, that was recently developed and approved for the treatment of GPP (15).

Indeed, current guidelines for GPP management include the use of conventional systemic drugs as first-line therapies (i.e., corticosteroids, cyclosporine, and methotrexate), small molecules, and biologic agents, that are mostly approved for the treatment of plaque psoriasis only. A very limited data provided solid evidence about their effectiveness in the treatment of pustular psoriasis, resulting in a partial control of the disease (16–20). The therapeutic unmet need related to the partial and unsatisfactory control of pustular psoriasis through the use of unapproved drugs is usually based on experts’ opinions or referring to physician-oriented assessment. Indeed, no studies investigated treatment satisfaction or included any evaluation through patient-reported outcomes (PROs) related to residual disease manifestations during systemic therapy. This cross-sectional, single-center, cohort-based study aimed to investigate patient satisfaction to treatment, quality of life of patients, and residual disease manifestations in pustular psoriasis patients undergoing treatment with systemic agents.

## Materials and methods

This cross-sectional, cohort-based study was performed at the Dermatology Unit of the Fondazione Policlinico Universitario A. Gemelli, Rome, Italy. Patients affected by pustular psoriasis, including GPP, Acrodermatitis continua of Hallopeau, and PPP, who referred to the dermatology outpatient clinic from May to July 2023, were included.

We selected patients treated for at least 16 weeks with any systemic therapy as monotherapy or combined with topical and/or systemic therapeutics. For all patients, we collected the following clinical and demographic data: age, sex, age of onset, disease duration, clinical phenotypes, concomitant comorbid conditions, topographical distribution of skin lesions, previous and current therapies, age at treatment initiation, duration of current treatment. Disease severity was assessed by Generalized Pustular Psoriasis Area and Severity Index (GPPASI) and Palmo Plantar Pustular Psoriasis Area and Severity Inde (PPPASI) score for GPP and PPP, respectively (21). The abovementioned measures score from 0, which indicates absence of clinical manifestations, to 72, which refers to a high grade of the disease (21). Additionally, the investigator global assessment (IGA) was used to measure the overall disease severity. IGA consists of a 5-point rating system, evaluating the presence of erythema, scale, and induration throughout the patient’s entire body, and rates disease severity on a scale from 0, absence of clinical signs, to 4, indicating a severe disease (22). Similarly, patient global assessment (PGA) enables an overall assessment of disease severity from patient perspective, reflecting the IGA score system (17).

Symptoms such as pain and pruritus were assessed using numerical rating scales (NRS), measuring the intensity of patient’s symptoms. The NRS typically ranges from 0 (no symptoms) to 10 (severe condition).

To assess patient’s quality of life (QoL) impairment deriving from the skin condition and patient satisfaction to treatment, three questionnaires were administered.

Short Form-36 (SF36) questionnaire consists of 36 questions that investigate eight different health domains, each representing a specific aspect of an individual’s health: physical functioning (PF), role limitations due to physical health (RP) and due to emotional health (RE), social functioning (SF), mental health (MH), energy/fatigue (EF), Pain (P) and general health (GH). This questionnaire evaluates health in each of these domains using a Likert scale. Through a dedicated scoring software, responses to questionnaire were analyzed obtaining values ranging from 0 to 100, with higher scores indicating better health and wellbeing (23).

DLQI, or Dermatology Life Quality Index, assesses the impact of skin conditions on a person’s quality of life (24). Ten questions investigating patient’s daily life, emotions, and activities are designed to be answered on a scale from 0 to 3, with 0 representing no impact on life quality and 3 representing a highly influencing disease severity. Higher DLQI scores thus indicate a greater impairment in the patient’s quality of life due to their skin condition (24).

To assess patient’s satisfaction to systemic therapies the TSQM (Treatment Satisfaction Questionnaire for Medication), a self-reporting questionnaire designed to assess patient satisfaction with specific medications or treatments, was administered (25). It covers four key domains, evaluating effectiveness, side effects, convenience, and global satisfaction, and typically uses a Likert scale ranging from “very satisfied” to “very dissatisfied.” For each domain, scores were dissected into 4 categories as grade of satisfaction: < 66%, 67–74%, 75–99% and 100%. Version II of TSQM have been used (25).

The present study was conducted in accordance with the Declaration of Helsinki initially published in 1964 on Ethical Principles for Medical Research Involving Human Subjects and the study protocol was reviewed and approved by Fondazione Policlinico Universitario Agostino Gemelli IRCCS–Università Cattolica del Sacro Cuore, Prot N.: 0002604/22—Prot. ID 4705. All subjects provided their written informed consent.

### Statistical analyses

Descriptive statistics was performed to analyze demographic and clinical variables. Categorical variables were described as frequencies and proportion, while continuous variables as mean ± standard deviation. Pearson correlation coefficient measured the strength of a linear association between disease severity, treatment satisfaction and global patients’ health scoring. Analysis of variance (ANOVA) was used to identify differences in the mean values of continuous variables among more than two groups.

Logistic regression models were designed to evaluate the association between global treatment satisfaction and physician-rated disease severity.

Statistical analysis was performed using the Stata/BE statistical package version 17 (StataCorp., TX, USA). Results were considered statistically significant with a *p*-value of < 0.05.

## Results

### Clinical and demographic features of patient cohort

A total of 31 patients (11 males and 20 females) with a mean (± SD) age of 56.4 (± 16.5) affected by pustular psoriasis and in treatment with biologic agents were included in the study. Seven patients (22.6%) were affected by GPP and 24 patients (77.4%) suffered from PPP. Out of 31, 11 (35.5%) and 9 (29.0%) patients had a history of plaque psoriasis and psoriatic arthritis, respectively. Fifteen patients (48.4%) had at least one comorbidity. Mean (± SD) disease duration was 15.0 (12.3) years.

At the time of observation, all patients underwent treatment with a biologic agent, revealing a mean (± SD) treatment duration of 33.0 (4.2) months. Overall, 14 (45.2%), 8 (25.8%), 6 (19.3%) and 3 (9.7%) patients were on treatment with an anti-interleukin (IL)-17, an anti-p19IL-23, an anti-TNFα and anti-p40IL12/23 agent, respectively (Table 1). In detail, among GPP patients, 4 out of 7 patients (57.1%) underwent therapy with IL- 17-, 2 (28.6%), TNFα-, and 1 (14.3%) p40IL12/23- inhibitors. PPP patients were mostly treated with an IL-17 (10/24, 41.7%), followed by p19IL-23 (8/24, 33.3%), TNFα (4/24, 16.7%), and anti-p40IL12/23 (2/24, 8.33%) inhibitors. Prior to current therapy, 25 out of 31 patients (80.6%) were previously treated with a conventional treatment, 16 (51.6%) were previously exposed to at least a biologic agent either as first-line therapy or not, and 6 patients (19.3%) were naïve to systemic therapies.

**TABLE 1:** Clinical and demographic characteristics of the whole patient cohort, and of the two PPP and GPP clinical variants, separately.

Clinical and demographic features	Overall (*n* = 31)	GPP (*n* = 7)	PPP (*n* = 24)	*p*-value
Age [mean (± SD)]	56.4 (16.5)	61.5 (10.6)	54.6 (17.7)	0.374
**Sex**				
Male	11 (35.5)	4 (57.1)	7 (29.2)	0.151
Female	20 (64.5)	3 (42.9)	17 (70.8)	
BMI [mean (± SD)]	26.7 (5.5)	24.0 (5.1)	27.6 (5.5)	0.136
**Comorbidities [*n* (%)]**				
Tot	15 (48.4)			
Cardiovascular	8 (53.3)	2 (28.6)	6 (25.0)	
Autoimmunity	3 (20.0)	1 (14.3)	2 (8.3)	0.782
Other	4 (26.7)	0 (0.0)	4 (16.7)	
**Current biological therapy [*n* (%)]**				
Anti-interleukin (IL)-17	14 (45.2)	4 (57.1)	10 (41.7)	
An anti-p19IL-23	8 (25.8)	0 (0.0)	8 (33.3)	0.360
An anti-TNFalfa	6 (19.3)	2 (28.6)	4 (16.7)	
Anti-p40IL12/23 agent	3 (9.7)	1 (14.3)	2 (8.33)	
**Previous biological therapy [*n* (%)]**				
Naïve	15 (48.4)	4 (57.1)	11 (45.8)	0.598
Bio experienced	16 (51.6)	3 (42.9)	13 (54.2)	

### Disease severity of patient cohort

At the time of observation, mean (± SD) GPPASI and PPPASI scores were 2.1 (01.9) and 2.0 (1.8), respectively. Out of 31, 13 (41.9%) patients achieved a clear or almost clear skin condition (IGA0-1; Table 2), without differences between GPP and PPP subcohorts (4/7, 57.1% vs. 9/24, 37.5%, *p* = 0.354, Table 3). Based on IGA score, 18 (58.1%) patients showed a moderate-to-severe form of pustular psoriasis, occurring in 62.5% (15/24) of PPP and 42.9% (3/7) of GPP patients (*p* = 0.354). On the contrary, based on PGA, 26 patients (83.9%) reported a severe or very severe skin condition, observed in 87.5% (21/24) of PPP and 71.4% (5/7) GPP cases (*p* = 0.309). Symptoms were assessed by NRS scales, reporting a mean (± SD) pruritus and pain intensity of 3.5 (1.4) and 2.8 (0.7), respectively.

**TABLE 2:** Disease severity scores of the whole patient cohort, and of the two PPP and GPP clinical variants separately.

Disease severity score	Overall (*n* = 31)	GPP (*n* = 7)	PPP (*n* = 24)	*p*-value
GPPASI [mean (± SD)]	-	2.1 (1.9)	-	0.583
PPPASI [mean (± SD)]	-	-	2.0 (1.8)	
DLQI [mean (± SD)]	8.6 (6.7)	8.8 (5.7)	8.8 (7.1)	0.981
**PGA[*n* (%)]**				
0–No disease	-	-	-	
1–Mild	-	-	-	
2–Moderate	5 (16.1)	2 (28.6)	3 (12.5)	
3–Severe	20 (64.5)	3 (42.9)	17 (70.8)	0.382
4–Very severe	6 (19.4)	2 (28.6)	4 (16.7)	
**IGA [*n* (%)]**				
0 No disease	6 (19.4)	1 (14.3)	5 (20.8)	
1 Mild	7 (22.6)	3 (42.8)	4 (16.7)	0.838
2 Moderate	9 (29.0)	2 (28.6)	7 (29.2)	
3 Severe	9 (29.0)	1 (14.3)	8 (33.3)	
4 Very severe	-	-	-	
**SF-36 [mean (± SD)]**				
Physical activity	66.7 (31.2)	63.3 (34.1)	66.4 (31.3)	0.834
Physical impairment	62.3 (36.1)	50 (27.4)	63.8 (37.8)	0.408
Emotional distress	57.1 (39.9)	49.5 (34.6)	57.3 (41.4)	0.674
Fatigue	55.3 (16.4)	56.8 (15.9)	55.0 (17.1)	0.814
Emotional wellbeing	53.4 (18.1)	56.7 (17.4)	52.0 (18.6)	0.581
Social functioning	70.3 (21.0)	70.8 (20.4)	71.0 (21.6)	0.986
Pain	67.2 (26.2)	62.9 (29.6)	67.8 (24.4)	0.694
Global TSQM score [mean (± SD)]	60.8 (19.3)	65.0 (59.6)	59.6 (20.6)	0.522
**Global TSQM ranges**				
100% *n* (%)	2 (6.5)	0 (0.0)	2 (8.3)	0.429
75–99% *n* (%)	11 (35.5)	3 (42.9)	8 (33.3)	0.643
74–66% *n* (%)	1 (3.2)	1 (14.3)	0 (0.0)	0.06
< 66% *n* (%)	17 (54.8)	3 (42.9)	14 (58.3)	0.469

**TABLE 3:** Quality of life and treatment satisfaction scores according to clinical disease variants.

Quality of life and treatment satisfaction assessment	Generalized pustular psoriasis (*n* = 7)	Palm-plantar pustular psoriasis (*n* = 24)	*P*-value
DLQI [mean (± SD)]	2.1 (5.7)	1.4 (7.1)	0.736
TSQM for global satisfaction [mean (± SD)]	65.0 (14.4)	59.6 (20.6)	0.522
SF36 for general health [mean (± SD)]	49.3 (18.1)	49.7 (20.1)	0.957
IGA 0/1 [*n* (%)]	4 (57.1)	9 (37.5)	0.354

In addition, mean (± SD) DLQI score was 8.6 (6.7). Out of 31 patients, 19 (61.3.%) had a DLQI of 5 or higher (15/24, 62.5%, in PPP subcohort versus 4/7, 57.1%, in GPP subcohort; *p* = 0.798), whereas only 6 patients presented DLQI of 0–1 within the PPP subcohort (6/24, 25%, of PPP patients versus 0/7, 0%, of GPP patients; *p* = 0.141) The impairment of general health was assessed through the SF36 questionnaire, scoring 50.4 (± 19.6) as global health status. Considering mean (± SD) values for SF36 individual items, the most affected domain was “*fatigue*” that scored 55.3 (16.4), followed by “*emotional wellbeing*,” scoring 53.4 (18.1). The mean (± SD) score for “*Emotional distress*” was 57.1 (39.9), for “*physical activity*” was 66.7 (31.2), and for “*limitations due to physical impairment*” was 62.3 (36.1). In addition, “*social functioning*” scored 70.3 (21.0) and “*pain*” was rated at 67.2 (26.2), thus indicating a lower degree of impact for both items.

Overall, TSQM score reflecting the “*global satisfaction*” was 60.8 (± 19.3; Table 2).

**FIGURE 1:**
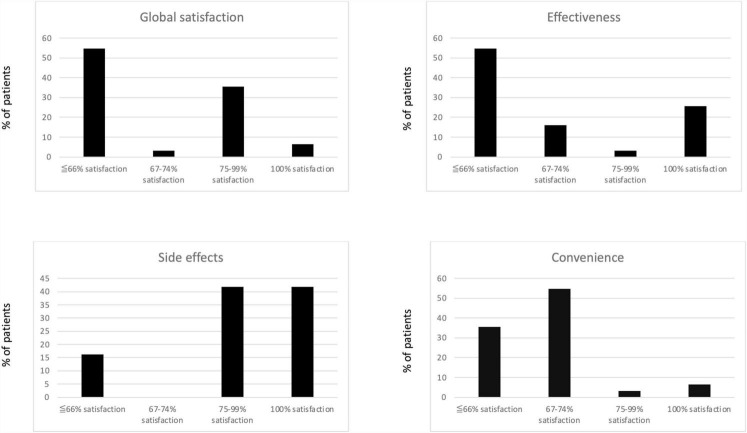
Scoring for all TSQM items.

Only two patients (2/31, 6.5%) achieved a full “*global satisfaction*” to treatment (TSQM score: 100%), 35.5% (11/31) of participants scored between 75–99%, and 3.2% (1/31) reported a level of “*global satisfaction*” falling into the 67–74% range (Figure 1). Poor “global satisfaction” (scores below 66%) were detected in 54.8% (17/31) of patients (Figure 1).

Among the TSQM items, we found a mean (± SD) score for “*effectiveness*” of 63.8 (24.6), “*side effects*” was rated as 81.4 (18.2), and “*convenience*” as 63.3 (16.9). Specifically, for the TSQM “effectiveness” item, eight out of 31 patients (25.8%) scored 100%, 3.2% (1/31) ranged between 75–99%, 16.2% (5/31) between 67–74%, while 54.8% (17/31) reported low satisfaction, scoring below 66%. As for “side effects,” 41.9% (13/31) of patients scored each 100% and 75–99%. Participants expressing lower satisfaction scores related to “side effects” were 16.2% (5/31). Finally, only 6.5% (2/31) reported a 100% score for the TSQM “convenience” item, 3.2% (1/31) and 54.8% (17/31) of patients reported a satisfaction grade comprised between 75–99% and 67–74%, respectively. In 35.5% (11/31) of patients a satisfaction grade below 66% was measured (Figure 1).

Dissecting the two subcohorts of patients affected by either GPP or PPP, we found no significant differences between the two patient subcohorts in terms of quality of life, measured either through the DLQI score or SF36 questionnaire, and treatment satisfaction, considering either as a mean score or clinically meaningful ranges (Table 3). Notably, DLQI 0–1 (19.4% of the population) were scored only among PPP cases, thus confirming the poor quality of life that characterized GPP patients (12, 26).

### Impact of residual disease manifestations on patients’ quality of life and satisfaction to treatment

Treatment Satisfaction Questionnaire for Medication (TSQM) global satisfaction scores was significantly affected by the residual disease severity during treatment. Patients with IGA2 or 3 reported a significant lower TSQM scoring values than patients with IGA0/1 (Prob *F* = 0.0013, R-squared = 0.44, Number of observations = 31). Consistently, positive TSQM global satisfaction (scored between 75 and 100%) was correlated with a six-fold higher likelihood to have a physician global assessment (IGA) of 0/1 (OR = 7.47, 95% CI = 1.39–39.8, *p* = 0.019), than negative TSQM global satisfaction (scored < 66%), independently from the clinical variant of pustular psoriasis (Table 4).

**TABLE 4:** Logistic regression model to derive the influence of treatment satisfaction on the probability to reach a PGA 0/1.

PGA 0/1	OR	Std. err.	*P*-value	[95% conf. interval]
Global TSQM < 66%	ref			
Global TSQM 66–74%	1 (empty)			
Global TSQM 75–100%	7.44	6.41	0.020	1.374–40.296
Palmoplantar PSO	ref			
Generalized PSO	1.97	2.05	0.515	0.255–15.197

Treatment Satisfaction Questionnaire for Medication (TSQM) global satisfaction score showed negative correlation with disease severity scores (GPPASI and PPPASI) (rho −0.58, *p* = 0.0006), similarly to SF36 global health score (rho −0.41, *p* = 0.022). By sub-dividing the study population according to the clinical variant of pustular psoriasis, the negative linear correlation between TSQM global satisfaction/SF36 global health and the clinical scores was confirmed only for PPP (TSQM: rho = −0.69, *p* = 0.0001; SF36:rho = −0.47, *p* = 0.012) and not for GPP (TSQM: rho = −0.17, *p* = 0.722; SF36:rho = −0.30, *p* = 0.509; Table 5).

**TABLE 5:** Analysis of variance (ANOVA) to identify the influence of disease severity on treatment TSQM scoring.

TSQM global	Coefficient	Std. err.	*P* > | t|	[95% conf. interval]
IGA0	ref			
IGA1	−11.92	8.506	0.172	−29.381 to 5.524
IGA2	−24.17	8.058	0.006	−40.701 to 7.632
IGA3	−34.33	8.058	< 0.0001	−50.967 to 17.89

Number of observations = 31, Prob > *F* = 0.0013, R-squared = 0.44.

## Discussion

The management of pustular psoriasis is reported as suboptimal, based on physician perspective whereas the evaluation of patient treatment satisfaction and the impact of residual disease manifestations on patients’ quality of life during treatment remained to be determined. No PROs were considered to better define this therapeutic unmet need in the treatment of pustular psoriasis. This is the first report assessing satisfaction, perception of disease severity and quality of life of patients during biologic therapies. In our patient cohort, all patients were treated with biologic agents including those targeting TNF-α, IL-17 and IL-23 that might obtain beneficial effects on the treatment of pustular psoriasis (27, 28), though only a few of them own the labeled indication for GPP (namely brodalumab, ixekizumab, and risankizumab in Japan). Beside the biologic agents having indication for GPP, acitretin, a systemic retinoid, is the only conventional agent approved by the FDA for both generalized and localized forms of pustular psoriasis (26, 29–34). Our study revealed an overall poor treatment satisfaction (TSQM below 66%) to these biologic therapies in most (54.8%) of pustular psoriasis patients undergoing treatment, with only two patients (6.5%) being fully satisfied (TSQM score equal to 100%) and a limited proportion (35.5%) achieving an elevated treatment satisfaction (TSQM score of at least 75%). Of note, the only two patients achieving 100% TSQM score were both PPP cases, while similar percentage of PPP and GPP patients reached at least 75% of TSQM score (33.3% for PPP and 42.9 for GPP). The described TSQM scoring for pustular psoriasis patients revealed a mixed perceptions, with a relatively low percentage of patients (41.9%) reported a high-grade (higher or equal to 75%) of overall satisfaction to treatment, suggesting that further improvements to enhance treatment satisfaction constituted a therapeutic unmet need.

This finding was associated with a residual disease activity that was assessed predominantly as moderate to severe (IGA 2–3 58.1% of patients), whereas based on patient perception disease severity was defined as severe or very severe in 83.9% of cases. Discrepancy between patients’ perception and physician’s assessment was also highlighted by the fact that 19.4% of patients resulted clear (IGA0) from skin lesions but no patients scored 0 or 1 for PGA. This represents further evidence suggesting how patient perspective on disease severity can differ significantly from physician evaluation. Also, residual disease manifestations resistant to current therapies, negatively influenced patients’ quality of life. In our study cohort, DLQI scoring revealed substantial proportion of patients experiencing a significant impairment in their quality of life due to their skin conditions, with 61.3% of patients having a DLQI score of 5 or higher, equally distributed among PPP and GPP cases. Conversely, DLQI 0-1 were detected only among PPP cases, confirming the poor quality of life that characterized GPP patients (12, 26).

Regarding the impairment of general health, the SF36 questionnaire detected limitations in physical activity and emotional distress, whereas the overall improvement obtained with the ongoing treatment reflected high SF36 scores in maintaining elevated social functioning scores.

In addition, we found a negative linear correlation between disease severity scores (assessed by GPPASI and PPPASI) and TSQM global satisfaction or SF36 global health, though it was significant only for PPP. However, differences between the two patient subcohorts were likely affected by the low number of GPP included in this study (*n* = 7), therefore it cannot be excluded that patients affected by GPP might have misperception of disease severity, expectations from the ongoing therapy, and psychological burden that differ from PPP patients.

Other studies investigated the psychometric validity of physician-based scoring systems (PGA and Psoriasis Area Severity Index (PASI)) in identifying residual disease after disease flares, notwithstanding the treatment prescribed (21), but no reports correlated the presence of residual disease during therapy with patient-reported treatment satisfaction and impact on quality of life, Our study suggest that an holistic evaluation of treatment response for pustular psoriasis is crucial and should include PROs to identify quiescent phases of disease that significantly affected patients’ quality of life.

Beside the strength of this study represented by the composite evaluation of disease severity, quality of life, and treatment satisfaction, there are some limitations related to the heterogeneity of treatments used in this cohort of patients and relatively limited number of patients included that affected the comparisons between GPP versus PPP subcohorts. In addition, the cross-section design of the study did not allow to investigate the occurrence of flares during treatment and their impact on patient quality of life and treatment satisfaction. Exploring the potential current challenges in preventing disease flares, that typically characterize pustular psoriasis, 72% of interviewed dermatologists considered available treatment options to be too slow to control flares (21). Along these lines, our findings suggested a relatively high impact of residual disease activity on patient satisfaction and quality of life confirming the high-need of novel and specific therapies, and standardized guidelines for the therapeutic management of pustular psoriasis, that should be considered a difficult-to-treat disease.

## Data availability statement

The original contributions presented in this study are included in this article/supplementary material, further inquiries can be directed to the corresponding author.

## Ethics statement

We confirm that all the subjects gave their written informed consent and the study protocol was reviewed and approved by Fondazione Policlinico Universitario Agostino Gemelli IRCCS–Università Cattolica del Sacro Cuore, Prot N.: 0002604/22—Prot. ID 4705.

## Author contributions

GCo: Data curation, Investigation, Writing – original draft. GF: Data curation, Funding acquisition, Writing – review & editing. GP: Data curation, Investigation, Writing – review & editing. EI: Visualization, Writing – review & editing. ED: Visualization, Writing – review & editing. NG: Visualization, Writing – review & editing. LD: Data curation, Formal analysis, Methodology, Writing – review & editing. GCa: Supervision, Visualization, Writing – review & editing. CD: Supervision, Writing – review & editing. AC: Conceptualization, Data curation, Formal analysis, Funding acquisition, Investigation, Methodology, Project administration, Resources, Software, Supervision, Validation, Visualization, Writing – review & editing. KP: Supervision, Validation, Visualization, Writing – review & editing.
